# SCEA-YOLO: A General-Purpose Maturity Grading Model of Multi-Crop Greenhouse Robots

**DOI:** 10.3390/plants15071102

**Published:** 2026-04-03

**Authors:** Tianyuan Li, Ping Liu, Dongfang Song, Xingtian Zhao, Xiangyu Lyu, Kun Zhang

**Affiliations:** 1 College of Mechanical and Electronic Engineering, Shandong Agricultural University, Tai’an 271018, China; 2 Shandong Key Laboratory of Intelligent Production Technology and Equipment for Facility Horticulture, Shandong Engineering Research Center of Agricultural Equipment Intelligentization, Shandong Agricultural University, Tai’an 271018, China; 3 State Key Laboratory of Wheat Breeding, Tai’an 271018, China

**Keywords:** maturity classification, instance segmentation, multi-crop learning, greenhouse agriculture, agricultural robots

## Abstract

Accurate classification of fruit maturity is essential for automated grading and robotic manipulation in modern greenhouse cultivation. Most existing methods rely on crop-specific models, severely restricting their scalability in multi-crop scenarios. To overcome this limitation, this study presents SCEA-YOLO, a unified and efficient instance segmentation framework built on YOLOv11s-seg, for simultaneous maturity classification of tomatoes and sweet peppers. To boost feature discrimination, reduce computational redundancy, and alleviate class imbalance, SCEA-YOLO integrates spatial-channel reconstruction convolution and an efficient multi-scale attention mechanism, while replacing the original detection head with the proposed EA-Head. The model is evaluated on a hybrid dataset captured under diverse greenhouse conditions, including varying illumination, fruit occlusion, and overlapping canopies. Its robustness to different viewing angles and camera distances is further validated via deployment on an automated grading robot. Compared with the baseline, SCEA-YOLO enhances classification precision and mAP_50–95_ by 5.3% and 2.3% for tomatoes, and 1.2% and 1.4% for sweet peppers, respectively. With only 33.2 GFLOPs, the model satisfies real-time inference demands. Benefiting from its lightweight structure and real-time performance, SCEA-YOLO can be readily deployed on embedded systems and robotic platforms. It offers a practical, unified, and scalable solution for intelligent fruit maturity evaluation in multi-crop greenhouse production.

## 1. Introduction

The increasing adoption of greenhouse cultivation has intensified the demand for automated and intelligent solutions to support crop monitoring, grading, and harvesting operations [1,2,3]. Tomatoes (*Solanum lycopersicum* L.) and sweet peppers (*Capsicum annuum* L.) represent two of the most economically significant horticultural crops in protected environments, distinguished by their high market value and extended harvesting cycles [4,5,6]. Fruit maturity serves as a pivotal indicator for determining optimal harvest timing, yield estimation, and post-harvest logistical planning [7,8]. Consequently, accurate and efficient maturity classification is critical for boosting production efficiency and mitigating labor dependency in modern greenhouse systems [9].

Conventional maturity assessment in greenhouse production predominantly relies on manual observation and experience-driven judgment [10,11]. This approach is not only labor-intensive but also susceptible to operator subjectivity and fatigue [12]. In recent years, computer vision and deep learning techniques have been increasingly deployed for fruit detection, segmentation, and maturity evaluation [13,14]. Convolutional Neural Network (CNN)-based frameworks, particularly the Mask R-CNN and You Only Look Once (YOLO) series, have demonstrated robust capabilities in extracting high-level visual features related to fruit color, morphology, and texture [15,16]. For instance, Sun and Wang proposed BMDNet-YOLO, a lightweight improved YOLOv8n model for field-grown blueberry maturity detection, which effectively mitigates the interference of occlusion, uneven illumination, and dense fruit distribution in unstructured agricultural environments through optimized feature extraction modules, a coordinate attention mechanism, and heterogeneous lightweight convolution design, achieving an excellent balance between detection accuracy and inference efficiency for edge deployment [17]. Nevertheless, most existing studies are confined to single-crop datasets and optimized for specific cultivation conditions, which results in poor generalization of the trained models and constrains their scalability in multi-crop greenhouse settings as well as their applicability for field deployment [18].

Automated maturity classification in real-world scenarios faces several key challenges [19]. Fruit ripening involves gradual visual changes, creating ambiguous inter-class boundaries and increasing misclassification during transitional stages [20]. Greenhouse environments further complicate perception due to dense foliage, fruit overlap, and uneven illumination [21,22]. Moreover, real-time deployment on edge devices with limited computational resources imposes strict constraints on model complexity and memory footprint [23]. These factors underscore the need for a classification framework that is both robust and efficient.

To address these limitations, recent studies have incorporated attention mechanisms, lightweight convolutional modules, and advanced loss functions to improve feature representation and training stability. Although such strategies enhance performance under controlled conditions, their evaluation is predominantly confined to single-crop scenarios. As a result, the potential of exploiting shared maturity-related visual features across phenotypically similar crops remains largely unexplored. Tomatoes and sweet peppers, which exhibit comparable color evolution patterns during ripening, provide a representative case for investigating the feasibility of a unified multi-crop maturity classification model.

In this study, SCEA-YOLO is proposed as an advanced instance segmentation framework for multi-crop maturity classification in greenhouse environments. Built on the YOLOv11s-seg backbone, the model integrates three complementary components: C3k2-ScConv for efficient spatial-channel feature reconstruction, Efficient Multi-Scale Attention (EMA) to enhance spatial discrimination under complex backgrounds, and an EA-Head to reduce computational overhead while improving robustness to multi-class imbalance. Together, these designs achieve a favorable balance between segmentation accuracy and real-time inference efficiency.

SCEA-YOLO introduces a unified framework for multi-crop maturity classification, trained on a hybrid dataset of tomato and sweet pepper images to enable cross-crop generalization. By integrating C3k2-ScConv, EMA, and EA-Head, the model achieves enhanced segmentation accuracy while maintaining computational efficiency for real-time edge deployment. The effectiveness of the proposed model is systematically validated via ablation experiments, benchmark comparisons with state-of-the-art (SOTA) instance segmentation frameworks, and field tests under varying imaging distances, viewing angles, and camera mounting heights. Experimental results demonstrate robust performance across crops, reliable generalization, and practical applicability in unstructured greenhouse environments. This work advances automated visual grading by providing a scalable, high-efficiency solution for multi-crop maturity perception, offering critical insights for intelligent greenhouse production systems and paving the way for general-purpose, real-time horticultural monitoring.

## 2. Materials and Methods

**Figure 1:**
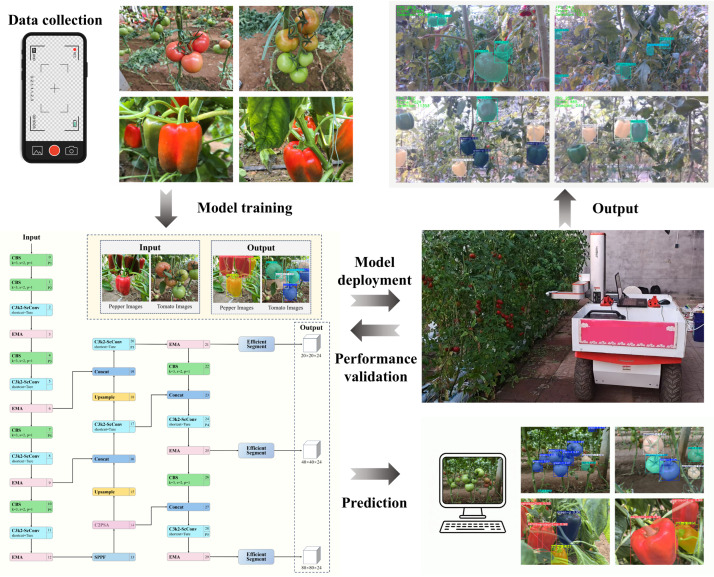
Overall workflow design.

With a view to addressing the requirements for maturity classification of tomatoes and sweet peppers in modern greenhouse environments, the SCEA-YOLO model was developed and deployed on a tomato-pepper (T-P) grading and detection robot. Model training, validation, and inference were conducted using the Ultralytics 8.4.11 framework to comprehensively evaluate detection performance. Furthermore, the practical infield performance of the model was validated through on-robot deployment experiments. The overall workflow of the proposed method is illustrated in Figure 1.

### 2.1. Data Collection

**Figure 2:**
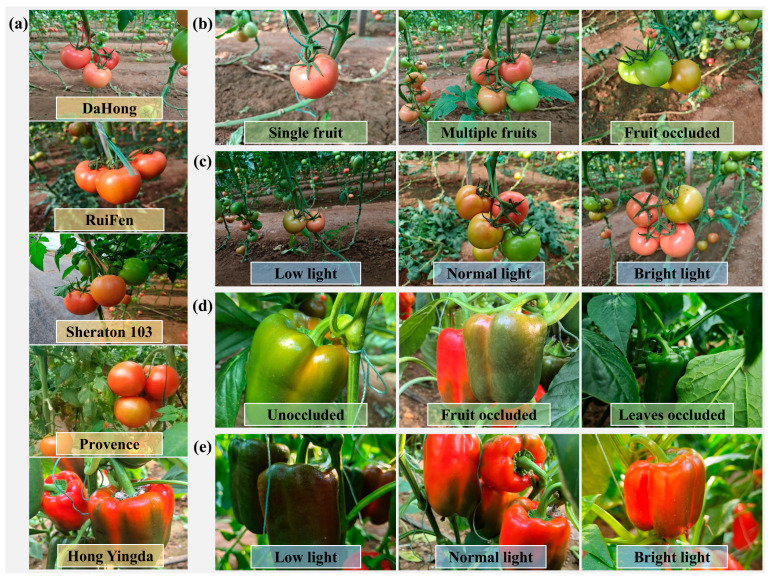
Tomato and sweet pepper datasets: (**a**) Varieties of tomatoes and sweet peppers; (**b**) Tomato images under multiple scenarios; (**c**) Tomato images in varying environments; (**d**) Sweet pepper images under different occlusion conditions; (**e**) Sweet pepper images in varying environments.

The dataset used in this study consists of images of tomatoes and sweet peppers collected between 9 November and 17 December 2024. Data acquisition was conducted at the Lushou Seed Industry Experimental Base in Xichen Village, Wenzhuang Subdistrict, Shouguang City, Shandong Province, China, as well as the Yuyi Company’s Shouguang Exhibition Greenhouse in Shouguang City, Shandong Province, China. To evaluate the generalization capability of the proposed model under real production conditions, the dataset (Figure 2) covers diverse illumination levels (low, normal, and high illumination) and occlusion scenarios, including non-occluded fruits, inter-fruit occlusion, and foliage occlusion.

### 2.2. Dataset Construction and Data Augmentation

Tomato and sweet pepper fruits were categorized into four maturity stages: Mature Green, Early Color Break, Late Color Break, and Fully Ripe. Dataset annotation was performed using the Image Segmentation Annotation Tool (ISAT). After filtering low-quality samples, a total of 3643 annotated images were retained. Among them, 2673 images were used for model training, while 970 images were divided into five independent groups for performance validation. The remaining unlabeled images were used exclusively for model inference. The detailed dataset composition is summarized in Table 1.

**Figure 3:**
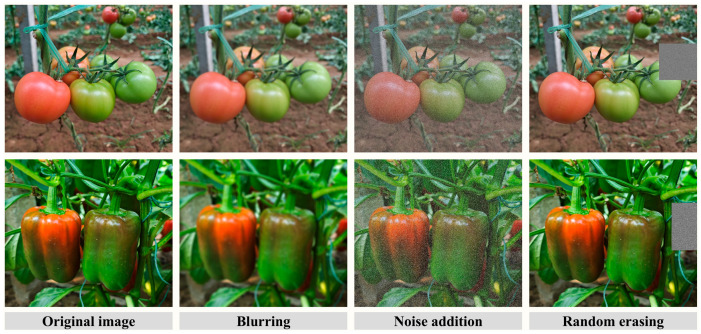
Data augmentation examples.

Offline data augmentation was applied to the training set to improve model robustness and mitigate overfitting. The augmentation strategies included blurring, Gaussian noise injection, and random erasing (Figure 3). After augmentation, a total of 6949 effective samples were obtained. The number of samples in each maturity category was balanced to approximately 2200, thereby reducing the adverse impact of class imbalance on model training [24].

### 2.3. SCEA-YOLO Model Architecture

**Figure 4:**
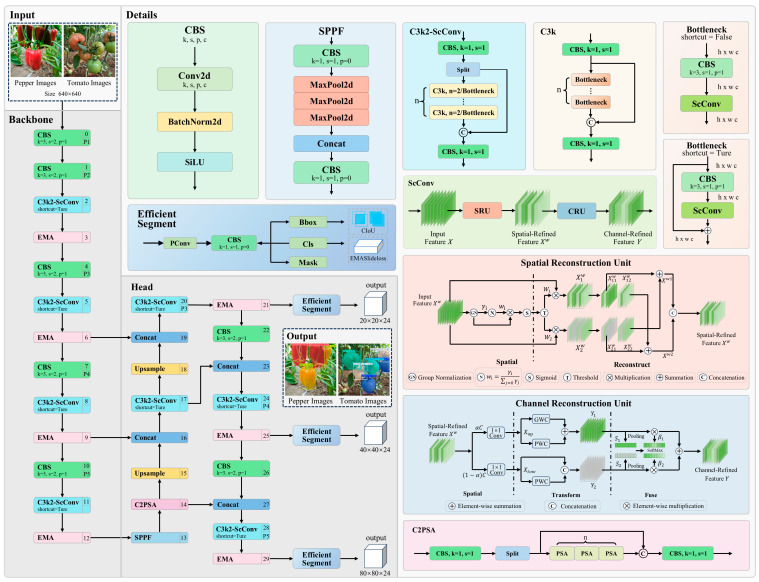
Overall architecture and key modules of the proposed SCEA-YOLO model.

The SCEA-YOLO model, constructed upon the YOLOv11s-seg baseline, is designed to achieve generalized maturity grading and detection for multiple crops. As illustrated in Figure 4, the architecture incorporates three strategic enhancements to address the challenges of unstructured greenhouse environments.

First, to optimize feature extraction efficiency, the model integrates the C3k2-ScConv module, which combines the C3k2 block with Spatial and Channel Reconstruction Convolutions (ScConv) [25]. By synergizing spatial and channel reconstruction units, this module effectively decouples informative features from redundant noise. This reconstruction process significantly reduces computational overhead while enhancing the representational capacity of both the backbone and neck networks, enabling SCEA-YOLO to process high-dimensional visual data efficiently in complex scenarios.

Second, the EMA mechanism is embedded to bolster spatial awareness and feature discrimination. EMA facilitates the aggregation of pixel-level pairwise relationships, capturing both large-scale global context and fine-grained local details [26]. This mechanism prevents interference from irrelevant scale features (e.g., background foliage) and ensures a balanced distribution of spatial semantics. Consequently, the model achieves higher target recognition accuracy with a reduced risk of overfitting to environmental noise.

Third, detection and segmentation performance is optimized via the EA-Head network. The EA-Head comprises two core components: an efficient detection head and the EMASlideLoss function.

Conventional detection heads typically follow an anchor-free paradigm, where two parallel convolutional branches are designated for bounding box regression and classification, respectively. In comparison, the Efficient Seg within the EA-Head is tailored for lightweight instance segmentation, which leverages PConv and CBS (1 × 1 convolution) modules for feature refinement and produces three parallel task-specific branches for bounding box regression, classification, and mask prediction, respectively. The lightweight architecture that significantly lowers parameter count and floating-point operations (FLOPs) without compromising accuracy [27].

Furthermore, EMASlideLoss replaces the standard BCEWithLogitsLoss. Compared with the widely adopted BCEWithLogitsLoss for binary classification and object detection, EMASlideLoss excels in mitigating sample imbalance. Under imbalanced positive-negative distributions with dominant easy samples, BCEWithLogitsLoss easy-sample gradients overwhelm parameter updates, causing deficient feature learning for hard samples such as small and occluded objects; moreover, it relies on manual hyperparameters for weighting adjustment, lacking adaptive capability. EMASlideLoss requires no manual tuning, dynamically partitioning easy-hard samples using the batch mean of prediction matching degree (e.g., IoU) as the adaptive threshold. Its slide-shaped nonlinear weighting mechanism amplifies hard-sample loss weights and suppresses easy-sample gradient interference, guiding the model to focus on critical samples. Retaining the numerical stability of BCEWithLogitsLoss, EMASlideLoss effectively resolves easy-hard sample imbalance and boosts model classification accuracy and robustness in complex scenarios.

### 2.4. Ablation Study Design

A comprehensive ablation study was designed to quantitatively assess the performance contributions of each architectural enhancement to the SCEA-YOLO model, thereby dissecting the individual efficacy and synergistic effects of the three core module optimizations (C3k2-ScConv, EMA and EA-Head). The experimental protocol for module combinations across all test cases is detailed in Table 2, with cases sequentially designated from the baseline model to the fully optimized SCEA-YOLO architecture to enable a stepwise performance analysis. YOLOv11s-seg was set as Case 1, serving as the baseline group to provide a benchmark for subsequent performance comparisons. Cases 2 to 4 were configured as single-module experimental groups, each incorporating one of the optimized modules independently. This design allowed for the isolated verification of the independent performance gains and functional validity of each modified module when integrated into the baseline framework. Building on this, Cases 5 to 7 were constructed as dual-module combination groups, where distinct pairs of the optimized modules were fused to explore the interactive synergies or potential performance trade-offs arising from the coupling of different module optimizations. Finally, all three core optimized modules were integrated together to form Case 8, which corresponds to the full SCEA-YOLO architecture. This ultimate experimental group enabled the validation of the overall performance of the complete design, as well as the comprehensive synergistic effects of the three modules on detection accuracy and computational efficiency for multi-crop maturity grading tasks.

### 2.5. Model Training Environment and Training Parameters

All model training and evaluation experiments were conducted on a workstation running the Windows 11 operating system. The deep learning environment was established using Python 3.10 and the PyTorch 2.7.1 framework, with CUDA 11.8 utilized for GPU-accelerated parallel computing. The hardware configuration comprised an AMD Ryzen 5 7600X3D 6-Core processor with a base frequency of 4.10 GHz (Advanced Micro Devices, Santa Clara, CA, USA) and an NVIDIA GeForce RTX 4070 Ti SUPER graphics processing unit (GPU) with 16 GB graphics video memory (VRAM) (NVIDIA Corporation, Santa Clara, CA, USA). To ensure reproducibility, the specific training hyperparameters and optimizer settings adopted for this study are detailed in Table 3.

### 2.6. Model Evaluation Metrics

To comprehensively evaluate the proposed model, a multi-dimensional metric system was established covering both detection accuracy and operational efficiency. Detection performance was quantified using Precision (*P*), Recall (*R*), mean Average Precision at IoU = 0.5 (mAP_50_), and mAP_50–95_. These metrics assess the model’s ability to correctly identify fruit targets and precisely localize their boundaries.

Simultaneously, the model’s suitability for deployment on resource-constrained robotic platforms was evaluated through three key efficiency indicators: number of parameters (Params) (Params) to measure model size, FLOPs to quantify computational complexity, and frames per second (FPS) to measure real-time inference speed. Collectively, these metrics provide a holistic assessment of the trade-off between grading accuracy and computational cost.

### 2.7. Model Deployment and Field Validation

**Figure 5:**
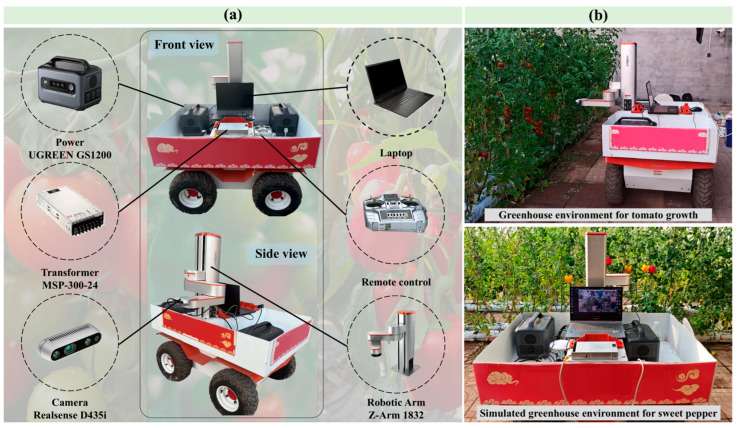
(**a**) Architecture of the T-P grading and detection Robot. (**b**) Shandong Agricultural University Protected Tomato Experimental Base.

In order to verify the practical efficacy of the proposed model, field validation experiments were conducted at the Facility Tomato Experimental Base of Shandong Agricultural University. A custom-developed T-P grading and detection robot served as the deployment platform for the general-purpose real-time grading system (Figure 5). Real-time grading detection was performed on tomato plants in an outdoor setting, while sweet pepper grading was validated via simulation testing using field-collected image data.

The robotic system hardware integrates a mobile chassis, a Z-Arm 1832 robotic manipulator, and an Intel RealSense D435i depth camera (Intel Corporation, Shenzhen, China) for visual perception. Power management is handled by a dedicated voltage regulation module and power supply unit. The core computing unit is a high-performance control laptop equipped with an Intel Core i9-14900HX CPU (Intel Corporation, Chandler, AZ, USA), 32 GB DDR5 memory (SK Hynix, Icheon, South Korea), and an NVIDIA GeForce RTX 4060 GPU (NVIDIA Corporation, Santa Clara, CA, USA), ensuring sufficient computational power for real-time inference.

**Figure 6:**
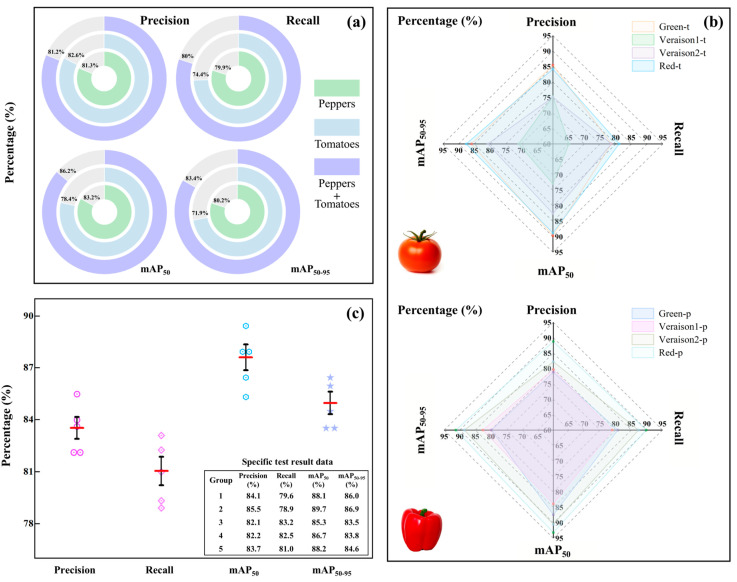
Performance evaluation of SCEA-YOLO: (**a**) Comparison of single-class and combined training effects; (**b**) Detection performance of tomatoes and sweet peppers; (**c**) Performance verification across multiple test sets.

So as to systematically evaluate the robot’s global detection performance and identify the optimal spatial configuration for field operations, experiments were conducted at the Protected Tomato Experimental Base of Shandong Agricultural University (Figure 6) across a range of spatial operation parameters. The camera posture was rigorously adjusted to test detection accuracy at five discrete heights, five observation angles, and five detection distances. This comprehensive testing protocol was designed to assess the model’s adaptability to the spatial variability inherent in tomato and sweet pepper plant structures.

## 3. Results and Discussion

### 3.1. Performance Evaluation of SCEA-YOLO

A mixed dataset comprising tomato and sweet pepper images was used for model training to explore the potential of multi-crop feature sharing and joint learning mechanisms. During training, strategic sampling and augmentation were employed to counter the long-tail distribution of maturity classes, thereby preventing the model from collapsing into trivial solutions for majority classes. Under multi-class and imbalanced data conditions, conventional cross-validation may introduce non-negligible uncertainty due to random partition bias; therefore, multiple independent test sets were adopted for a rigorous performance assessment.

For the purpose of evaluating the feasibility and theoretical advantage of mixed-crop training, model performance obtained from the combined tomato-pepper dataset was compared with that of models trained on single-crop datasets. According to the results presented in Figure 6a, tomatoes and sweet peppers show analogous visual maturation patterns, including surface texture and specular reflection properties. Consequently, mixed-dataset training exerted no negative impact on average detection accuracy. Instead, a notable increase in mAP_50–95_ was observed. Theoretically, the mixed dataset acts as a form of implicit data augmentation, introducing a regularization effect that forces the backbone network to learn more robust, invariant feature representations rather than memorizing crop-specific nois. This validates that the SCEA-YOLO architecture successfully captures the shared high-level semantic features (such as geometric shape and glossiness) across different solanaceous crops, demonstrating strong inductive transfer capability.

The tomato and sweet pepper datasets were categorized into four maturity stages: Mature Green, Early Color break, Late Color break,, Fully Ripe, corresponding to the four legends in Figure 6: Green, Veraison1, Veraison2, Red. For tomatoes, higher detection accuracy was achieved at distinct color stages (Green and Red), whereas performance declined during color transition stages, accompanied by an increased number of false positives [28]. From a feature space perspective, the “Early” and “Late” color-turning stages share highly coupled feature distributions, leading to blurred decision boundaries and semantic ambiguity. The SCEA-YOLO model, however, minimizes this intra-class variance through its enhanced attention mechanisms. For sweet peppers, lower detection accuracy was observed for green fruits. This presents a classic “Camouflage Object Detection” challenge, where the spectral characteristics of the target overlap significantly with the background foliage [29]. The slight performance drop indicates that solely relying on RGB information creates a bottleneck in feature discrimination when foreground-background contrast is low.

Performance fluctuations were observed on the validation set. However, this set was primarily used for parameter tuning and preliminary assessment. To further evaluate the model’s robustness and generalization capability, the test dataset was divided into five independent subsets with minimal distributional similarity.

Model performance was evaluated on each subset, and the average values were used as the final test results. It can be observed from Figure 6c that performance on the test sets exceeded that on the validation set, indicating stable generalization. This multi-subset testing strategy effectively eliminates the randomness associated with single-split validation, confirming that SCEA-YOLO has learned intrinsic morphological and chromatic features rather than overfitting to specific environmental biases.

### 3.2. Ablation Study Results

The ablation study results, presented in Table 4, quantify the individual and collective contributions of the C3k2-ScConv, EMA, and EA-Head modules to the model’s detection performance and computational efficiency. This analysis aims to decouple the orthogonal contributions of feature reconstruction, spatial attention, and efficient prediction heads within the SCEA-YOLO architecture.

In single-module experiments, replacing the baseline C3k2 with C3k2-ScConv (Case 2) reduced FLOPs to 32.1 G while increasing tomato detection precision by 3.5%. Theoretically, this improvement stems from the ScConv mechanism, which effectively separates spatial and channel redundancy from informative features. By suppressing noise and emphasizing representative feature maps, the model achieves a higher signal-to-noise ratio, leading to improved precision. Introducing the EMA module alone (Case 3) enhanced multi-scale feature representation, raising the tomato mAP_50_ to 84.8%. This suggests that EMA’s cross-spatial learning capability captures long-range dependencies and global context, which are critical for distinguishing fruits in dense, cluttered environments. Conversely, utilizing the EA-Head alone (Case 4) reduced FLOPs to 31.0 G but caused a slight decline in accuracy. This indicates that while the lightweight head reduces parameter count, its limited feature representational capacity creates a bottleneck when not supported by enhanced upstream feature extraction.

In multi-module configurations, the interaction between modules reveals complex architectural dynamics. The combination of C3k2-ScConv and EMA (Case 5) improved detection performance but increased computational cost, as the dense attention calculations of EMA added to the model’s load. Interestingly, the combination of EMA and EA-Head (Case 7) resulted in lower performance than the baseline, despite reduced FLOPs. This phenomenon can be attributed to a feature mismatch: the rich, multi-scale features generated by EMA may overwhelm the limited capacity of the lightweight EA-Head without the intermediate feature refinement and compression provided by ScConv, leading to information loss during the prediction phase.

When all three modules were integrated (Case 8, SCEA-YOLO), the model achieved a synergistic effect, reaching the optimal balance between accuracy and complexity. Compared to the baseline YOLOv11s, tomato Precision, Recall, mAP_50_, and mAP_50–95_ increased by 5.3%, 1.2%, 0.7%, and 2.3%, respectively. Corresponding improvements for sweet peppers were 1.2%, 0.3%, 0.4%, and 0.4%, with FLOPs reduced to 33.2 G. This configuration represents a Pareto-optimal solution for the grading task.

The results demonstrate a clear functional division: C3k2-ScConv acts as a feature purifier, enhancing the representation of color-transitioning fruits by reconstructing spatial and channel information; EMA serves as a semantic focuser, suppressing background noise via coordinate attention; and EA-Head ensures inference efficiency. Crucially, the high-quality feature maps produced by C3k2-ScConv compensate for the potential information loss in the lightweight EA-Head, ensuring that the reduction in computational cost does not compromise the model’s discriminative power.

### 3.3. Comparison with Mainstream Models

SIn orderto rigorously benchmark the proposed architecture against state-of-the-art paradigms, SCEA-YOLO was systematically compared with representative instance segmentation models. These baselines encompass three distinct architectural strategies: two-stage detectors (Mask R-CNN) [30], large-scale foundation models (SAM2-Tiny) [31], and one-stage detectors (YOLOv8s-seg, YOLOv9c-seg, YOLOv11s-seg, and YOLOv12s-seg). All models were evaluated under identical experimental settings to ensure a fair assessment of their feature encoding and generalization capabilities.

**Figure 7:**
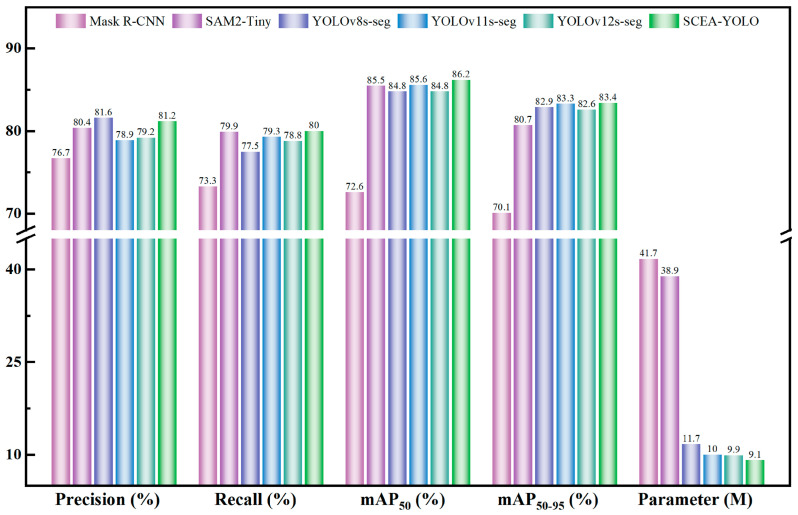
Performance comparison of SCEA-YOLO with various mainstream models.

As shown in Figure 7, SCEA-YOLO achieves superior or competitive performance across key metrics (P, R, mAP_50_, mAP_50–95_). Specifically, the model exhibits statistically significant gains in mAP_50–95_, a strict metric that penalizes poor boundary alignment. This indicates that SCEA-YOLO possesses stronger object localization and fine-grained boundary segmentation capabilities in complex scenarios. Theoretically, this advantage is derived from the C3k2-ScConv module, which mitigates feature aliasing during downsampling, allowing the model to preserve high-frequency edge information essential for delineating occluded fruits and subtle maturity transitions.

From the perspective of ccomputational efficiency the pnumber of parameterscount of SCEA-YOLO is significantly lower than that of Mask R-CNN and SAM2-Tiny, and lalso ower than comparable YOLO variants. While SAM2-Tiny benefits from massive pre-training, its generalized encoder carries redundant weights unnecessary for specific agricultural tasks, resulting in computational inefficiency. In contrast, SCEA-YOLO creates a compact feature space tailored for maturity classification. This reduction in model size is achieved without sacrificing performance, effectively demonstrating that domain-specific architectural optimization can outperform generalized foundation models in targeted applications. It can be seen from this that SCEA-YOLO achieves an optimal trade-off on the accuracy-efficiency Pareto frontier. This renders it highly suitable for the edge intelligence requirements of agricultural inspection robots, where high-precision inference must be executed within the strict latency and energy constraints of embedded hardware.

### 3.4. Eigen-CAM-Based Attention Visualization Analysis

To visually validate the internal decision-making process and semantic consistency of the proposed architecture, Eigen-CAM was employed for attention visualization. Eigen-CAM computes the first principal component of the spatial feature maps, which effectively captures the dominant structural patterns learned by the network without the noise introduced by backpropagation [32]. This characteristic makes it a reliable tool for analyzing feature learning mechanisms in agricultural vision models facing complex greenhouse background interference. Attention heatmaps were generated based on the final C3k2-ScConv layer in the neck network (corresponding to C3k2 in YOLOv11 and C2f in YOLOv8), with activation intensity graded from blue (low feature importance) to red (high feature importance), consistent with the standard visualization setting of Eigen-CAM.

**Figure 8:**
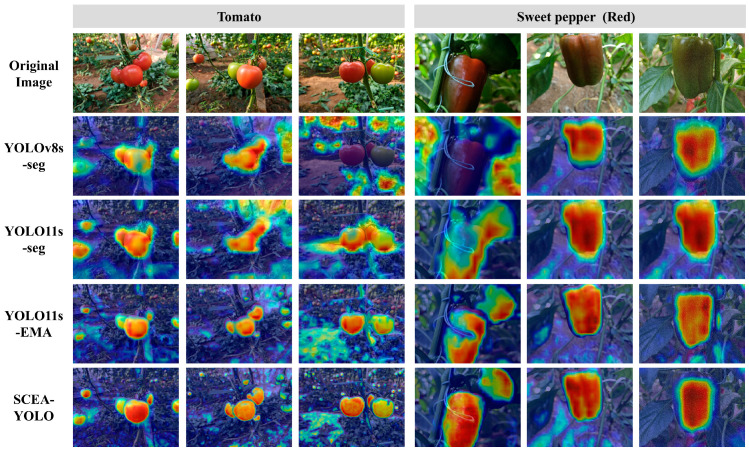
Eigen-CAM heatmap visualization results.

Comparative analysis of the heatmaps (Figure 8) reveals distinct feature activation patterns across models, which directly reflect the differences in their feature learning and discrimination capabilities. The original YOLOv8s-seg and YOLOv11s-seg baselines exhibit obvious feature drift: high-response regions frequently spill over from fruit targets to background foliage, especially in areas with similar texture or color to fruit peels. This indicates a lack of semantic concentration, leading to lower recall and precise boundary localization. This phenomenon indicates that the baseline models lack effective semantic screening of features, and easily confuse background redundant features with fruit maturity-related visual features, which is the core reason for their low detection precision in complex greenhouse environments. The YOLOv11s-seg integrated with the EMA mechanism shows improved focus, it still suffers from dispersion in complex scenes, which is due to insufficient upstream feature filtering, leading to the EMA mechanism amplifying both valid and redundant features of the target.

In contrast, SCEA-YOLO exhibits superior spatial-semantic alignment in its activation patterns. Heatmaps reveal that high-response activation regions are strictly confined to fruit contours, and irrelevant background regions are significantly suppressed. Notably, the model also displays consistent activation targeting for different maturity stages of the same crop and the same maturity stage across different crops. For both tomatoes and sweet peppers, SCEA-YOLO selectively activates the core visual feature regions associated with distinct maturity stages: green-mature fruits show focused activation on the non-occluded fruit body and calyx; color-turning fruits anchor high-response pixels to color-transition patches, which serve as the key basis for differentiating early and late color-turning stages; fully ripe fruits exhibit high-intensity activation across the entire fruit epidermis. This superior activation pattern arises directly from the synergistic interplay among the three core architectural modules of SCEA-YOLO, which constitutes a well-structured feature processing pipeline consisting of spatial-channel reconstruction, multi-scale semantic attention, and efficient balanced prediction. The C3k2-ScConv module decouples informative features from redundant noise via spatial and channel reconstruction, filters out background interference to generate high-signal-to-noise ratio feature maps, and fundamentally addresses raw feature pollution in baseline models to underpin accurate attention focusing. The EMA mechanism then aggregates pixel-level pairwise relationships and captures global context and local details based on these purified feature maps, enabling selective amplification of maturity-related visual features rather than simple spatial fruit localization, and overcoming the feature activation homogenization of the standalone EMA module. Finally, the EA-Head adjusts the weighting of difficult samples, effectively mitigating the effects of multi-class imbalance in maturity grading and ensuring the stability and consistency of activation patterns across all maturity stages and crop types.

From the perspective of image-based high-throughput plant phenotyping (HTPP), existing HTPP studies on fruit maturity classification mostly focus on the improvement of model numerical indicators, but lack in-depth exploration of the internal feature learning mechanism [33]. This study reveals the shared semantic feature learning pattern of solanaceous crops (tomatoes and sweet peppers) in maturity grading through Eigen-CAM visualization: the model prioritizes learning the color and texture evolution features related to fruit ripening rather than crop-specific shape features. This finding not only provides visual and interpretable evidence for the cross-crop generalization ability of SCEA-YOLO, but also offers a feasible research path for the development of general-purpose multi-crop phenotyping models, which is of great significance for the practical application of computer vision technology in intelligent greenhouse production.

### 3.5. Deployment Performance Evaluation

To ensure the engineering feasibility and robustness of the T-P general-purpose grading model in edge-computing scenarios, real-time detection experiments were conducted using the T-P grading and detection robot. System performance was systematically evaluated by adjusting detection distance, camera height, and pose angle to simulate the dynamic unstructured variables of real-world greenhouse conditions.

**Figure 9:**
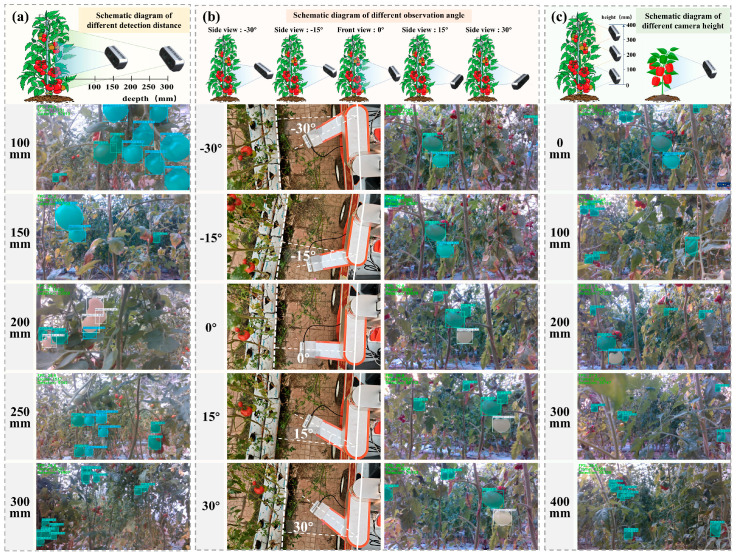
Detection Results of Field Deployment: (**a**) Grading performance under different detection distances; (**b**) Grading performance under different observation angles; (**c**) Grading performance under different camera heights.

The impact of detection distance, which governs the spatial resolution and feature granularity of the target, was first evaluated to validate the model’s scale invariance. Experiments were conducted at distances ranging from 100 mm to 300 mm, corresponding to varying fields of view (FOV). As shown in Figure 9a and Video S1, SCEA-YOLO demonstrated stable detection across all distances. Theoretically, this stability is underpinned by the EMA module’s cross-spatial learning capability, which effectively aggregates multi-scale context. This mechanism mitigates the “feature semantic gap” caused by drastic changes in object scale, ensuring that the model maintains high discriminative power for both high-resolution near-field targets and low-resolution far-field targets, thereby confirming the robot’s reliability in dynamic greenhouse settings.

Subsequently, the influence of observation angle was analyzed to determine viewpoint robustness, considering that camera orientation dictates geometric deformation and illumination consistency. Using 0° as the reference, performance was recorded at intervals between −30°, −15°, 0°, 15°, and 30° (Figure 9b). At 0°, minimal occlusion and uniform lighting yielded optimal performance. While slight specular highlights and occlusion at ±15° caused only minor accuracy fluctuations (1.2–2.5%), severe affine distortion of fruit features combined with occlusion at ±30° led to significant accuracy drops of 8.7–11.3%. These results demonstrate strong rotational robustness under small angular variations while defining the critical boundary conditions for the active vision system, providing a theoretical basis for optimizing path planning algorithms to maintain the camera pose within the effective recognition envelope.

Finally, vertical coverage was assessed to verify spatial consistency across the plant’s growth profile. Experimental groups were established at 100 mm vertical increments starting from the 0.6 m baseline, and detection performance validation was conducted across the height range of 0.6 m to 1.0 m. (Figure 9c and Video S2). At the baseline of 0.6 m, the robot successfully detected the lowest fruit layers, while top-layer fruits were effectively captured at 1.0 m. Performance remained stable across all heights, demonstrating the model’s invariance to background heterogeneity—specifically, its ability to distinguish fruits against soil backgrounds at lower levels versus skylight or structural backgrounds at upper levels. This confirms that the system’s operational range effectively covers the entire vertical canopy of tomato and sweet pepper plants.

The experiments confirmed that SCEA-YOLO exhibits favorable scale invariance and rotational robustness under small angular deviations, as well as strong invariance to background heterogeneity in the vertical canopy detection of crops, enabling full-range maturity grading detection of plant canopies. Geometric distortion and occlusion of fruit features caused by large-angle poses act as the core constraints leading to a significant decline in the model’s detection accuracy, and also define the critical boundaries for the path planning of the vision system. When a laptop equipped with an RTX 4060 graphics card is employed as the controller, the recognition time for each target is within 16 ms. Meanwhile, affected by device latency, the FPS of the camera interface remains in the range of 28.2 to 29.6. This translates to a more real-time response with the adoption of a mini host boasting higher computing power, which also demonstrates that the model possesses practical engineering application value for the field deployment of multi-crop maturity grading in greenhouses and can satisfy the real-time inference requirements of agricultural robots. Nevertheless, the large-angle pose constraint and the performance bottleneck in detecting low-contrast/camouflaged targets remain urgent problems to be solved in practical application, which requires optimizing path planning and fusing multi-modal data to enhance the model’s adaptability to field environments [34].

## 4. Conclusions

This study presentsSCEA-YOLO, a lightweight, high-precision instance segmentation deep learning architecture specifically developed for unified maturity classification of tomatoes and sweet peppers in complex unstructured greenhouse environments. By strategically integrating the C3k2-ScConv module, the EMA mechanism, and the EA-Head network, the framework achieves a synergistic optimization of feature extraction and computational efficiency. From a theoretical perspective, these architectural enhancements effectively decouple spatial and channel redundancy while enforcing long-range semantic dependencies, thereby addressing the “feature ambiguity” caused by complex backgrounds and subtle phenotypic variations in maturity. Quantitative evaluations on a mixed-crop dataset demonstrate the model’s strong inductive transfer capability, validating that shared maturity-related features can be effectively learned across different solanaceous crops without crop-specific retraining. Compared to the YOLOv11s-seg baseline, SCEA-YOLO achieved a 5.3% improvement in Precision and a 2.3% increase in mAP_50–95_ for tomatoes, and 1.2% and 1.4% increases respectively for sweet peppers. Crucially, the model maintains a low computational footprint of 33.2 GFLOPs, representing an optimal trade-off on the accuracy-efficiency Pareto frontier that satisfies the strict latency constraints of agricultural mobile robots. Furthermore, field deployment experiments on the T-P Intelligent Grading Robot confirmed the system’s engineering feasibility, demonstrating robust performance stability under varying detection distances, observation angles, and vertical heights. Visualization analysis via Eigen-CAM further corroborates that the model successfully suppresses background noise and focuses on intrinsic fruit features, ensuring high segmentation integrity even under occlusion. Future research will prioritize extending this framework to a broader range of crop species to explore the limits of cross-domain generalization.

## Figures and Tables

**Table 1:** Dataset composition and sample distribution.

	Images	Train (80%)	Val (10%)	Test (10%)				
				1	2	3	4	5
Tomato	2299	1510	189	190	189	191	189	190
Bell Pepper	1344	864	110	110	111	109	111	110
Total	3643	2374	299	300	300	300	300	300

**Table 2:** Ablation experiment design.

Improved Models	Module			
	Yolov11s	C3k2-ScConv	EMA	EA-Head
Case1	√	—	—	—
Case2	√	√	—	—
Case3	√	—	√	—
Case4	√	—	—	√
Case5	√	√	√	—
Case6	√	√	—	√
Case7	√	—	√	√
Case8 (SCEA-YOLO)	√	√	√	√

**Table 3:** Model training parameters.

Parameters	Setting
Epoch	150
Workers	4
Batch size	8
Optimizer	AdamW
Input image size	640 × 640
Initial learning rate	0.0001
Final learning rate	0.01

**Table 4:** Ablation study results of SCEA-YOLO.

	Class	Precision (%)	Recall (%)	mAP_50_ (%)	mAP_50–95_ (%)	F1 Score (%)	FLOPs (G)
Case 1 (YOLO11s)	Tomato	76.5	74.7	82.8	79.0	75.6	33.1
	Pepper	81.2	83.9	88.4	84.2	82.5	
Case 2	Tomato	80.0	73.9	83.5	79.8	76.8	32.1
	Pepper	82.4	83.2	88.8	84.6	82.8	
Case 3	Tomato	76.8	74.6	84.8	81.0	75.7	31.5
	Pepper	82.2	81.5	88.6	82.8	81.8	
Case 4	Tomato	76.2	74.8	81.3	78.1	75.5	31.0
	Pepper	80.4	84.1	87.0	82.6	82.2	
Case 5	Tomato	79.5	76.7	83.9	79.5	78.1	35.3
	Pepper	82.5	80.9	89.4	85.2	81.7	
Case 6	Tomato	81.6	78.4	83.6	82.1	80.0	34.2
	Pepper	82.4	83.2	88.8	84.6	82.8	
Case 7	Tomato	78.5	74.3	82.8	78.7	76.3	30.1
	Pepper	85.2	83.4	85.4	84.1	84.3	
Case 8 (SCEA-YOLO)	Tomato	81.8	75.9	83.5	81.3	78.7	33.2
	Pepper	82.4	84.2	88.8	85.6	83.3	

## Data Availability

The data are not publicly available due to privacy. The datasets generated during and/or analyzed during the current study are available from the corresponding author upon reasonable request.

## Supplementary Materials

The supporting information can be downloaded at: https://www.mdpi.com/article/10.3390/plants15071102/s1.
